# The Association of Medical Preoperative Evaluation Using Clinical Video Telehealth With Hospital Length of Stay: Descriptive Analysis

**DOI:** 10.2196/38054

**Published:** 2022-07-25

**Authors:** Brittany Nicole Burton, Sara Arastoo, Simon Wu, Nancy Liu, Michael K Ong, Sondra Vazirani

**Affiliations:** 1 Department of Anesthesiology and Perioperative Medicine University of California Los Angeles Health Los Angeles, CA United States; 2 Department of Medicine Veterans Affairs Greater Los Angeles Healthcare System Los Angeles, CA United States

**Keywords:** telemedicine, telehealth, eHealth, digital health, hospital, length of stay, veteran's health, video, veteran, preoperative, outpatient, chart review, retrospective, clinical care, effectiveness, efficacy, discharge

## Abstract

**Background:**

Preoperative medical evaluation serves to identify risk factors and optimize patients before surgery. Providing a telehealth option in the perioperative setting has played a significant role in reducing barriers to quality perioperative health care.

**Objective:**

We aimed to evaluate how telemedicine preoperative evaluations using Clinical Video Telehealth (CVT) impact hospital length of stay.

**Methods:**

We performed a retrospective chart review between 2016 and 2017 of adult patients who underwent evaluations in our hospitalist-run preoperative medicine clinic. Patients seen in our preoperative CVT program were compared to patients seen in person to evaluate the association of visit type (preoperative CVT versus in-person evaluation) with hospital length of stay, defined as hospital stay from postoperative day 0 to discharge. There were 62 patients included in this retrospective study.

**Results:**

The adjusted incidence rate ratio (IRR) for hospital length of stay was significantly shorter in patients who underwent preoperative CVT compared to an in-person visit (IRR 0.52, 95% CI 0.29-0.92, *P*=.02).

**Conclusions:**

After adjusting for age and comorbidities, we show that preoperative telemedicine in the perioperative setting is associated with a shorter hospital length of stay compared to in-person visits. This suggests that telemedicine can play a viable role in this clinical setting.

## Introduction

For the almost 50 million surgeries and procedures performed annually in the United States, preoperative medical evaluation serves to identify and optimize perioperative risk to decrease adverse outcomes and to prevent same-day cancellations of surgery [1]. Traditionally, preoperative evaluations are performed face-to-face in the clinic. Starting in July 2014, given its large catchment area, the Veterans Affairs Greater Los Angeles Healthcare System implemented a telemedicine preoperative medicine clinic using Clinical Video Telehealth (CVT). CVT is a technology that Veterans Affairs (VA) providers have used since the early 2000s. With CVT, clinicians can gather relevant history and conduct a limited physical exam using a camera and digital stethoscope. Since adoption, CVT has found increasing rates of use, especially for patients living in rural areas, who face significant barriers to completing in-person visits. In the wake of the COVID-19 pandemic, the importance of providing a telehealth option in outpatient care has become even more apparent [2].

Several studies in non-VA settings have demonstrated that preoperative evaluations done via telemedicine are associated with high patient/provider satisfaction, cost savings, and a lower rate of same-day cancellation when compared to in-person evaluations [3-6]. However, the potential limitations of telemedicine preoperative evaluation (eg, not performing a comprehensive physical exam may preclude clinical diagnoses) may lead to subsequent case-cancellation complications. Thus, our project aimed to evaluate how telemedicine preoperative evaluations using CVT impact hospital length of stay.

## Methods

### Overview

This manuscript follows the Strengthening the Reporting of Observational Studies in Epidemiology (STROBE) Statement guidelines for reporting observational studies. All data used in this study were extracted from electronic medical records.

We performed a retrospective chart review of adult patients who underwent evaluations in our hospitalist-run preoperative medicine clinic. Patients seen in our preoperative CVT program, which started in July 2014, were compared to patients who had in-person visits to evaluate the association of visit type (preoperative CVT versus in-person) with hospital length of stay, defined as hospital stay from postoperative day 0 to discharge. We extracted data from 2016 to 2017. Preoperative CVT involves a thorough history and a full airway exam. Exclusion criteria for the CVT preoperative program were defined at the program’s start as American Society of Anesthesiologists (ASA) class 4, or ASA class 3 and uncontrolled blood pressure (>180/100 mm Hg) and/or diabetes (glycated hemoglobin [HbA_1c_] >9%). The patients needed to meet all criteria to be recommended for an in-person visit and therefore be excluded from CVT. These patients were recommended for in-person evaluation due to comorbidity burden and importance of taking a complete history and conducting a physical exam.

### Data Analysis

Statistical analysis was performed using R (version 3.6.1; R Foundation for Statistical Computing). To measure the differences in hospital length of stay among those who received CVT versus face-to-face consultation, chi-square and student *t* tests were used. Multivariable negative binomial regressions were performed, adjusting for age, gender, ASA score, surgery type (major or minor), and Elixhauser comorbidity index. The incidence rate ratio (IRR), 95% CIs, and *P* value were calculated for each estimate.

### Ethical Considerations

Our study was reviewed by the Institutional Review Board of the West Los Angeles Veterans Administration Medical Center and was granted an “exempt” status.

## Results

There were 62 patients included in this retrospective study. The cancellation rate was 1.74% for CVT versus 3.48% for in-person. Table 1 outlines the distribution of patient characteristics stratified by preoperative visit type. In this unadjusted analysis, there were no significant differences between the cohorts.

Table 2 outlines the negative binomial regression for the association of visit type with hospital length of stay. The age- and Elixhauser score–adjusted incidence rate for hospital length of stay was significantly shorter in patients who underwent preoperative CVT compared to an in-person visit (IRR 0.52, 95% CI 0.29-0.92, *P*=.02).

**Table 1:** Participant characteristics.

Characteristics			In-person (n=29)		Preoperative Clinical Video Telehealth (n=33)	*P* value^a^	
Age (years), mean (SD)			62.83 (11.23)		59.36 (15.43)	.32	
**Gender, n (%)**							.26
	Male	28 (96.6)		28 (84.8)			
	Female	1 (3.4)		5 (15.2)			
**Elixhauser comorbidity score, n (%)**							.16
	≤1	5 (17.2)		11 (33.3)			
	0	6 (20.7)		9 (27.3)			
	≥1 and <5	8 (27.6)		10 (30.3)			
	≥6 and <10	4 (13.8)		1 (3)			
	≥11 and <19	6 (20.7)		2 (6.1)			
**Surgical specialty, n (%)**							.68
	Urology	7 (24.1)		6 (18.2)			
	Colorectal	1 (3.4)		2 (6.1)			
	Ophthalmology	1 (3.4)		0 (0)			
	Plastic surgery	1 (3.4)		5 (15.2)			
	General	6 (20.7)		3 (9.1)			
	Orthopedics	6 (20.7)		6 (18.2)			
	Gynecology	0 (0)		1 (3)			
	Ear, nose, and throat	2 (6.9)		4 (12.1)			
	Neurosurgery	4 (13.8)		5 (15.2)			
	Vascular	1 (3.4)		1 (3)			
**ASA class, n (%)^b^**							.13
	1	1 (3.4)		2 (6.1)			
	2	0 (0)		5 (15.2)			
	3	27 (93.1)		24 (72.7)			
	4	1 (3.4)		2 (6.1)			
	Length of stay, mean (SD)	6.55 (9.09)		3.33 (3.97)		.07	

^a^Pearson chi-square test for categorical variables. Student *t* test for continuous variables.

^b^ASA: American Society of Anesthesiologists.

**Table 2:** The association of preoperative visit with hospital length of stay.

	Incidence rate ratio	95% CI	*P* value
Preoperative Clinical Video Telehealth^a^	0.52	0.29-0.92	.02
Elixhauser comorbidity	1.00	0.82-1.22	.27
Age	1.01	0.98-1.03	.98

^a^Reference group for preoperative Clinical Video Telehealth is patients who received medical chart review and did not receive preoperative Clinical Video Telehealth.

## Discussion

In summary, we show that preoperative CVT, while holding age and the Elixhauser comorbidity score constant in the model, has an IRR for hospital length of stay that is 0.52 times lower compared to in-person visits. This study found a significant difference in the IRR of postoperative length of stay between patients receiving telehealth versus in-person preoperative evaluations. This suggests that telemedicine can play a viable role in this clinical setting. Telemedicine has the potential to increase care access across all specialties and health care systems. Our findings had several limitations including that the study was retrospective, was conducted at a single center, and had a low sample size, leading to an increased risk of type II error. Length of stay may be affected by many factors. In the VA patient population, social reasons may affect length of stay more than the typical patient population. There likely is selection bias between those patients who were willing to do CVT versus those who wanted an in-person evaluation.

In our patient population, several patients were more interested in telemedicine compared to in-person visits and we hope to expand to other locations. We plan to apply biomedical informatics to the electronic medical records to extract granular patient data including but not limited to (1) demographic data (age, race, socioeconomic status, and zip code), (2) comorbidities and severity of each comorbidity, (3) postoperative complications, (4) telemedicine-specific data (cancellation rates, missed appointments, and scheduling delays), and (5) patient perceptions and experiences. We hope this research design will help us to identify the benefits and potential disadvantages of telemedicine in the perioperative period. Future studies should be prospective and adequately powered to limit type II error. In addition, future studies should explore how to appropriately triage patients as being “telehealth-appropriate” in the preoperative setting, as well as investigate the effects of preoperative telehealth on other patient-centered outcomes.

## Abbreviations

ASA: American Society of Anesthesiologists
CVT: Clinical Video Telehealth
IRR: incidence rate ratio
STROBE: Strengthening the Reporting of Observational Studies in Epidemiology
VA: Veterans Affairs
